# Supplementary material for: Machine Learning-Driven Classification of Urease Inhibitors Leveraging Physicochemical Properties as Effective Filter Criteria
Source: Int J Mol Sci. 2024 Apr 13;25(8):4303. doi: 10.3390/ijms25084303 (PMC11049951; doi:10.3390/ijms25084303)
Supplement: Supplementary file 1 [file ijms-25-04303-s001.zip › supplementary_information_IJMS.pdf]

International Journal of *Molecular Sciences*

MDPI

Article

Machine Learning-Driven Classification of Urease Inhibitors Leveraging Physicochemical Properties as Effective Filter Criteria

Natalia Morales [1], Elizabeth Valdés-Muñoz [2], Jaime González [1], Paulina Valenzuela-Hormazábal [3], Jonathan M. Palma [4], Christian Galarza [5], Ángel Catagua-González [5], Osvaldo Yáñez [6], Alfredo Pereira [7,*] and Daniel Bustos [8,*]

1 Magíster en Ciencias de la Computación, Universidad Católica del Maule, Talca 3460000, Chile; nmoralesr@ucm.cl (N.M.); jaime.gonzalez@alu.ucm.cl (J.G.)
2 Doctorado en Biotecnología Traslacional, Centro de Biotecnología de los Recursos Naturales, Universidad Católica del Maule, Talca 3480094, Chile; elizabeth.valdes@alu.ucm.cl
3 Departamento de Farmacología, Facultad de Ciencias Biológicas, Universidad de Concepción, Concepción 4030000, Chile; paulinvalenzuela@udec.cl
4 Facultad de Ingeniería, Universidad de Talca, Curicó 3344158, Chile; jonathan.palma@utalca.cl
5 Departamento de Matemáticas, Facultad de Ciencias Naturales y Matemáticas, Escuela Superior Politécnica del Litoral, Guayaquil EC090903, Ecuador; chedgala@espol.edu.ec (C.G.); anglucat@espol.edu.ec (Á.C.-G.)
6 Núcleo de Investigación en Data Science, Facultad de Ingeniería y Negocios, Universidad de las Américas, Santiago 7500000, Chile; oyanez@udla.cl
7 Facultad de Ingeniería, Arquitectura y Diseño, Universidad San Sebastián, Bellavista 7, Santiago 8420524, Chile
8 Laboratorio de Bioinformática y Química Computacional, Departamento de Medicina Traslacional, Facultad de Medicina, Universidad Católica del Maule, Talca 3480094, Chile
* Correspondence: alfredo.pereira@uss.cl (A.P.); dbustos@ucm.cl (D.B.)

Citation: Morales, N.; Valdés-Muñoz, E.; González, J.; Valenzuela-Hormazábal, P.; Palma, J. M.; Galarza, C.; Catagua-González, Á.; Yáñez, O.; Pereira, A.; Bustos, D. Machine Learning-Driven Classification of Urease Inhibitors Leveraging Physicochemical Properties as Effective Filter Criteria. *Int. J. Mol. Sci.* **2024**, *25*, 4303. https://doi.org/10.3390/ijms25084303

Academic Editor: Dong-Jun Yu

Received: 15 March 2024
Revised: 3 April 2024
Accepted: 8 April 2024
Published: 13 April 2024

Copyright: © 2024 by the authors. Licensee MDPI, Basel, Switzerland. This article is an open access article distributed under the terms and conditions of the Creative Commons Attribution (CC BY) license (https://creativecommons.org/licenses/by/4.0/).

Table S1. Physicochemical descriptors obtained from rCDK program.

Feature name	Description
**Topological parameters**	
ECCEN	Calculation that integrates distanceand adjacency information.
fragC	Calculate the complexity of a molecule
Kier1 Kier2 Kier3	Calculate the Kier and Hall kappa molecular shape indices, which are descriptors used to quantify the shape of molecules. These indices provide a numerical value that represents the overall structural characteristics of a molecule.
khs.sLi khs.ssBe khs.ssssBe khs.ssBH khs.sssB khs.ssssB khs.sCH3 khs.dCH2 khs.ssCH2 khs.tCH khs.dsCH khs.aaCH khs.sssCH khs.ddC khs.tsC khs.dssC khs.aasC khs.aaaC khs.ssssC khs.sNH3 khs.sNH2 khs.ssNH2 khs.dNH khs.ssNH khs.aaNH khs.tN khs.sssNH khs.dsN khs.aaN khs.sssN khs.ddsN khs.aasN khs.ssssN khs.sOH khs.dO khs.ssO khs.aaO khs.sF khs.sSiH3 khs.ssSiH2 khs.sssSiH khs.ssssSi khs.sPH2 khs.ssPH khs.sssP khs.dsssP khs.sssssP khs.sSH khs.dS khs.ssS khs.aaS khs.dssS khs.ddssS khs.sCl khs.sGeH3 khs.ssGeH2 khs.sssGeH khs.ssssGe khs.sAsH2 khs.ssAsH khs.sssAs khs.sssdAs khs.sssssAs khs.sSeH khs.dSe khs.ssSe khs.aaSe khs.dssSe khs.ddssSe	Compute the occurrences of the E- state fragments.

khs.sBr khs.sSnH3 khs.ssSnH2 khs.sssSnH khs.ssssSn khs.sI khs.sPbH3 khs.ssPbH2 khs.sssPbH khs.ssssPb	
MDEC-11 MDEC-12 MDEC-13 MDEC-14 MDEC-22 MDEC-23 MDEC-24 MDEC-33 MDEC-34 MDEC-44 MDEO-11 MDEO-12 MDEO-22 MDEN-11 MDEN-12 MDEN-13 MDEN-22 MDEN-23 MDEN-33	Calculate the molecular distance edge descriptors, which are used to assess the distances between specific atoms in a molecule. In the case of carbon (C), nitrogen (N), and oxygen (O) atoms, these descriptors can provide valuable information about the connectivity and spatial arrangement of these elements within a molecule.
PetitjeanNumber	Calculate the Petitjean Number, which is a descriptor used to quantify the topological complexity of a molecule. It provides a numerical value that represents the branching and connectivity of atoms within a molecular structure.
TopoPSA	Compute the topological polar surface areabased on fragment contributions.
WTPT-1 WTPT-2 WTPT-3 WTPT-4 WTPT-5	Calculate the weighted path (molecular ID) descriptors, as described by Randic, are used to characterize the branching patterns within a molecule. These descriptors assign weights to the paths between atoms in a molecular graph, reflecting the importance of each path in terms of molecular branching.
WPATH WPOL	The Wiener path number and Wiener polarity number are descriptors used to quantify the topological complexity and polarity of a molecule, respectively. The Wiener path number calculates the sum of the distances between all pairs of atoms in a molecular graph. It provides a measure of the overall molecular size and branching. On the other hand, the Wiener polarity number evaluates the sum of the distances between pairs of atoms in the molecular graph, considering the type of bond connecting them. It characterizes the polar nature of a molecule by considering the differences in electronegativity between bonded atoms.
Zagreb	The sum of the squared atom degrees of all heavy atoms is a descriptor used to measure the complexity and connectivity of heavy atoms in a molecule.
**Electronic parameters**	

Apol	Calculate the sum of the atomic polarizabilities, including implicit hydrogens, is a measure of the overall electron cloud distortion capability of a molecule.
Bpol	Calculate the sum of the absolute value of the difference between the atomic polarizabilities of all bonded atoms in the molecule, including implicit hydrogens, quantifies the variation in electron cloud distortion between bonded atoms
PPSA-1 PPSA-2 PPSA-3 PNSA-1 PNSA-2 PNSA-3 DPSA-1 DPSA-2 DPSA-3 FPSA-1 FPSA-2 FPSA-3 FNSA-1 FNSA-2 FNSA-3 WPSA-1 WPSA-2 WPSA-3 WNSA-1 WNSA-2 WNSA-3 RPCG RNCG RPCS RNCS THSA TPSA RHSA RPSA	A list of descriptors that combine partial charge information with surface area
nHBAcc	Hydrogen bond acceptors
nHBDon	Hydrogen bond donors
	**Constitutional parameters**
nA nR nN nD nC nF nQ nE nG nH nI nP nL nK nM nS nT nY nV nW	
naAromAtom	Number of amino acids
nAtom	Number of aromatic atoms
nB	Number of atoms
nAtomLC	Number of bonds
nAtomP	Number of atoms located in the longest chain
nRotB	Number of atoms located in the longest π chain
LipinskiFailures	Number of non-rotatable bonds
MW	Number of violations of Lipinski's Rule Of Five
XLogP	Molecular Weight
	Partition coefficient
	**Hybrid parameters**
BCUTw-1l BCUTw-1h BCUTc-1l BCUTc-1h BCUTp-1l BCUTp-1h	
Wlambda1.unity Wlambda2.unity Wlambda3.unity Wnu1.unity Wnu2.unity Wgamma1.unity Wgamma2.unity Wgamma3.unity Weta1.unity Weta2.unity Weta3.unity WT.unity WA.unity WV.unity WK.unity WG.unity WD.unity	Eigenvalue based descriptors used in chemical diversity described by Pearlman et al.

	**Holistic descriptors described by Todeschini et al.**
	**Geometrical parameters**
GRAV-1 GRAV-2 GRAV-3 GRAVH-1 GRAVH-2 GRAVH-3 GRAV-4 GRAV-5 GRAV-6	
LOBMAX LOBMIN	Characterizes the mass distribution
MOMI-X MOMI-Y MOMI-Z MOMI-XY MOMI-XZ MOMI-YZ MOMI-R	Calculates the proportional relationship between the length and breadth dimensions of a molecule
topoShape geomShape	Compute the principal moments of inertia and ratios of the principal moments.
	Calculate the topological and geometric shape indices described by Petitjean and Bath et al. respectively, are measures that assess the anisotropy or shape asymmetry of a molecule.

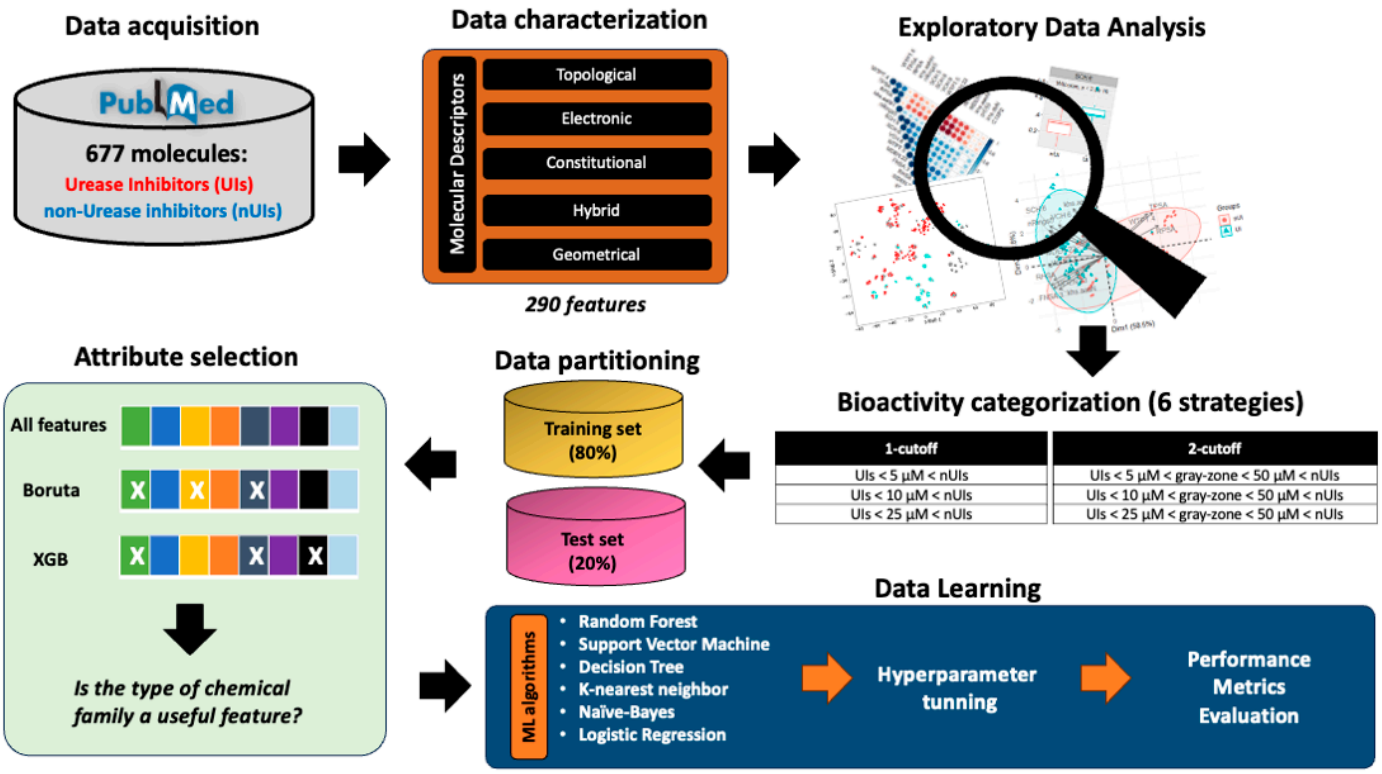

Figure S1. Schematic workflow. Each stage of the protocol is represented as follow: 1) Data acquisition, 2) Data characterization, 3) Exploratory Data Analysis, 4) Bioactive categorization, 5) Data partitioning, 6) Attribute selection, and 7) Data Learning.

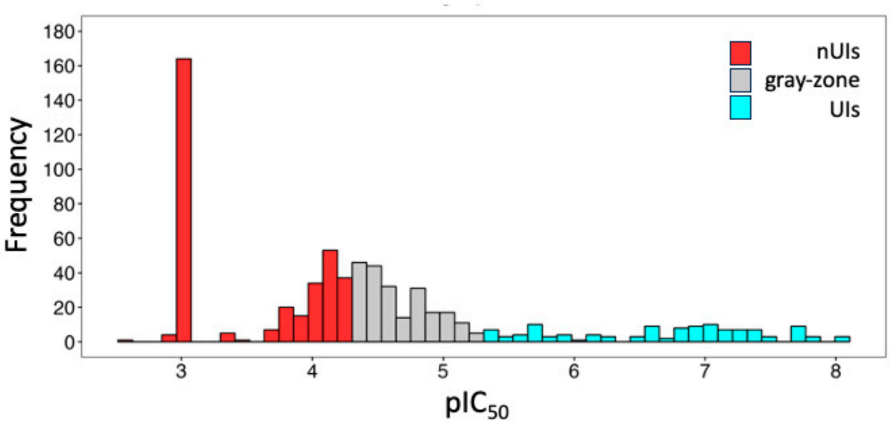

Figure S2. Distribution of the dataset based on bioactive characterization. The histogram illustrates the frequency of the three labeled groups: non-urease inhibitors (nUIs) in red, urease inhibitors (UIs) in cyan, and molecules falling within the 4.3 > pIC_{50} < 5.3 range, referred to as the gray-zone, shown in gray.

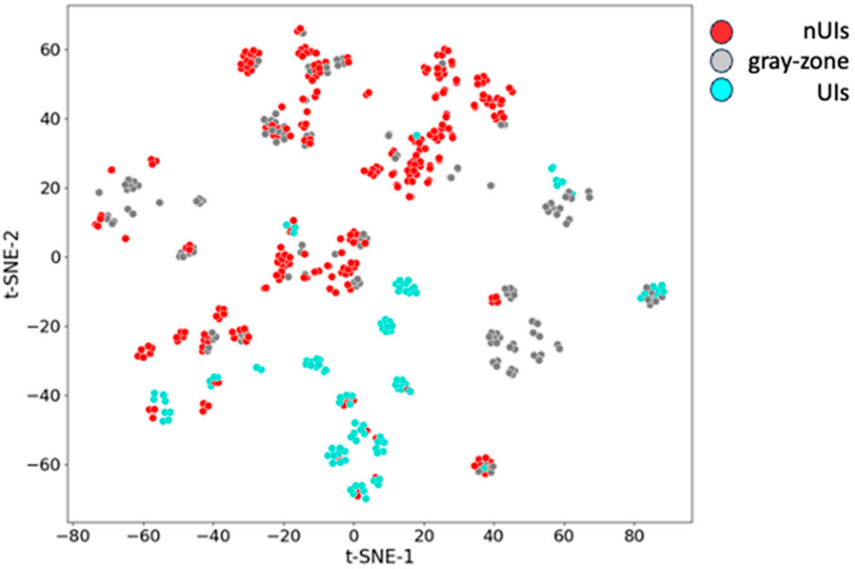

Figure S3. t-SNE dimensional map, where each data point represents a distinct compound categorized as a urease inhibitor (UIs) indicated in cyan, encompassing molecules exhibiting an IC_{50} ≤ 5 µM. Similarly, non-urease inhibitors (nUIs) are denoted in red, encompassing molecules with an IC_{50} ≥ 50 µM. Additionally, compounds existing in the gray-zone, defined by IC_{50} values falling within the range of 5 > IC_{50} < 50, are shaded in gray.

Disclaimer/Publisher's Note: The statements, opinions and data contained in all publications are solely those of the individual author(s) and contributor(s) and not of MDPI and/or the editor(s). MDPI and/or the editor(s) disclaim responsibility for any injury to people or property resulting from any ideas, methods, instructions or products referred to in the content.
